# Decreased Netrin-1 in Mild Cognitive Impairment and Alzheimer’s Disease Patients

**DOI:** 10.3389/fnagi.2021.762649

**Published:** 2022-02-16

**Authors:** Ting Ju, Lina Sun, Yuwei Fan, Tianhang Wang, Yanchen Liu, Dan Liu, Tianyi Liu, Chang Zhao, Wenxin Wang, Lijun Chi

**Affiliations:** ^1^Department of Neurology, The First Affiliated Hospital of Harbin Medical University, Harbin, China; ^2^Intensive Care Unit, Jiangyin People’s Hospital, Wuxi, China; ^3^Department of Neurology, Shenzhen Samii Medical Center, Shenzhen, China

**Keywords:** Alzheimer’s disease, mild cognitive impairment, netrin-1, IL-17, TNF-α, neuroinflammation

## Abstract

**Background and Objective:**

Inflammatory mediators are closely associated with the pathogenesis of Alzheimer’s disease (AD) and mild cognitive impairment (MCI). Netrin-1 is an axon guidance protein and despite its capacity to function as a neuroimmune guidance signal, its role in AD or MCI is poorly understood. In addition, the association among netrin-1, cognitive impairment and serum inflammatory cytokines such as interleukin-17 (IL-17) and tumor necrosis (TNF-α) remains unclear. The aim of this study was to determine serum levels of IL-17, TNF-α and netrin-1in a cohort of AD and MCI patients, and to study the relationship between these cytokines and cognitive status, as well as to assess the possible relationships between netrin-1 levels and inflammatory molecules.

**Methods:**

Serum concentrations of netrin-1, TNF-α and IL-17 were determined in 20 AD patients, 22 MCI patients and 22 healthy controls using an enzyme-linked immunosorbent assay (ELISA). In addition, neuropsychological evaluations and psychometric assessments were performed in all subjects.

**Results:**

Serum netrin-1 levels were decreased in AD and MCI patients and were positively correlated with Mini Mental State Examination (MMSE) scores. In contrast, serum TNF-α and IL-17 levels were elevated in AD and MCI cohorts and negatively correlated with MMSE scores. Serum netrin-1 levels were inversely related with TNF-α and IL-17 levels in AD, but not MCI, patients.

**Conclusion:**

Based on the findings reported here, netrin-1 may serve as a marker for the early recognition of dementia and predict cognitive impairment.

## Objective

Alzheimer’s disease (AD) is an age-related, prevalent neurodegenerative disorder, with gradual memory loss and behavioral impairments manifest as the most salient symptoms (McKhann et al., 1994, Mckhann et al., 2011; Tarawneh and Holtzman, 2012). According to the 2015 World Alzheimer Report, over 46 million people currently live with dementia worldwide, a number which is estimated to peak at 131.5 million in 2050 (Prince, 2015). Mild cognitive impairment (MCI) is recognized as a transition phase between normal cognition and a clinical diagnosis of probable dementia in individuals not yet meeting the criteria for dementia, but at a greater risk of developing this condition (Petersen et al., 1999; Etgen et al., 2011). Approximately 10-15% of individuals with MCI develop dementia every year, compared with 1-2% in unaffected individuals (Levey et al., 2006; Petersen, 2010).

Netrin-1, an endogenously secreted laminin-related protein, is identified as a bifunctional neuronal guidance molecule through its interactions with canonical receptors (Rajasekharan and Kennedy, 2009). Results from a number of studies have demonstrated that netrin-1 inhibits migration of monocytes, neutrophils and lymphocytes via activation of its receptors, which then contributes to its unique effects upon immune responses (Ly et al., 2005; Aherne et al., 2013). Recent clinical research findings have revealed that there is a marked down-regulation of netrin-1 in various inflammatory and autoimmune diseases (Liu et al., 2016; Mulero et al., 2017; Chen J.-L. et al., 2019; Bruikman et al., 2020). In accordance with these clinical findings are results from animal models (e.g., rats, mice and porcine) demonstrating that an administration of recombinant netrin-1 improves organ function, reduces leukocyte infiltration and suppresses production of cytokines and chemokines (Wu et al., 2008; Mutz et al., 2010; Tadagavadi et al., 2010; Liao et al., 2013; Ranganathan et al., 2013a; Wang et al., 2017).

Amyloid-β (Aβ)-induced neurotoxicity has been largely accepted as a hallmark component in the pathogenesis of AD, because Aβ accumulates in extracellular deposits to form the senile plaques observed in these patients. Results from previous studies have shown that netrin-1, via its interaction with amyloid precursor protein (APP, from which Aβ, the main component of the amyloid plaques associated with AD is derived) (Lourenço et al., 2009; Rama et al., 2012; Borel et al., 2017), suppresses Aβ peptide production as demonstrated both *in vivo* and *in vitro*. As a result of netrin-1 treatment, there are improvements in cognitive dysfunction in animal models of AD and a prevention of Aβ-induced cell death in AD cell models and Aβ-induced oxidative stress and neuroinflammation (Lourenço et al., 2009; Spilman et al., 2016; Zamani et al., 2019, 2020). Within our laboratory, decreases in netrin-1 were correlated with a Th17/Tregs (T helper 17/regulatory T cells) balance disorder in a rat model of Aβ-induced AD (Sun et al., 2019). When the findings of these studies are collated, they suggest netrin-1 administration may represent an appealing strategy that can improve the memory and neuronal loss associated with AD.

The exact etiology of AD remains unknown, but collective results from biochemical and neuropathological investigations have suggested that neuroinflammation plays a fundamental role in the pathogenesis and development of AD and MCI (McGeer et al., 1987; Rogers et al., 1988; Akiyama et al., 2000; Miguel and Maria, 2012; Eldik et al., 2016). Increasing evidence has been presented indicating that inflammatory pathways are activated within pathologically susceptible regions in the brains of AD patients (e.g., entorhinal, temporoparietal and cingulate cortex), effects which are accompanied with high levels of inflammatory mediators (e.g., pro-inflammatory cytokines and chemokines) in AD, as well as MCI, patients (Hye et al., 2006; Sperling et al., 2011). Such amplifications of cytokines and accumulations of complement factors have been shown to be implicated in AD-related memory loss and declines in learning capability and mental activity.

The impact of pro-inflammatory cytokines on the etiopathogenesis of AD has been exhaustively studied, with IL-1, IL-6, IL-10, IL-12, IL-17, IL-18, IL-23, tumor necrosis factor-α (TNF-α), interferon-γ (IFN-γ) and vascular endothelial growth factor (VEGF), all being associated with neuroinflammatory processes (Guillot-Sestier et al., 2015; Chen J.-M. et al., 2019; Alvarez et al., 2020; He et al., 2020; Leko et al., 2020). Moreover, these augmentations in pro-inflammatory cytokines have also been associated with the cognitive impairments observed in AD patients (Galimberti et al., 2008; Holmes et al., 2009; Lai et al., 2017). In this study, levels of TNF-α and IL-17 were determined as references for inflammatory cytokine responses. IL-17 is a pro-inflammatory cytokine secreted by the newly described CD4+ helper T cell subset, Th17 cells. Results from previous *in vivo* and *in vitro* studies have provided evidence indicating that elevated levels of IL-17 are present in both cerebrospinal fluid and serum of AD patients (Doecke et al., 2012; Chen et al., 2014). Such findings suggest that this Th17 cell subset may be involved with the inflammatory responses associated with this disease. In support of this suggestion were the observations that increased serum levels of IL-17 were found in AD patients as compared with levels obtained in controls (Doecke et al., 2012; Chen et al., 2014).

TNF-α plays an important role in the induction and maintenance of inflammation in the central nervous system and elevations in TNF-α have been found in tissue, cerebrospinal fluid and serum of both patients and animal models of inflammatory and autoimmune pathologies, including AD. In fact, TNF-α may exert potent effects upon amyloidosis and neurodegeneration, along with learning and memory deficits in AD and, cognitive decline may be more rapid in patients with high levels of TNF-α (Holmes et al., 2009).

As these studies described above suggest a potentially important role of netrin-1 in neuroinflammation and thus AD, the aim of this study was to first determine serum levels of netrin-1, IL-17 and TNF-α in a cohort of AD and MCI patients. We then examined the relationship between netrin-1 levels and cognitive status as a means to assess possible associations among netrin-1 levels, inflammatory molecules and AD/MCI.

## Materials and Methods

### Patients

#### Cognitive Status Evaluation and Diagnosis

Neuropsychological evaluations and psychometric assessments were performed using a Neuropsychological Battery including the Mini Mental State Examination (MMSE), Neuropsychiatric Inventory (NPI), clinical dementia rating (CDR), the Hachinski Ischemic Scale and Alzheimer disease assessment scale-cognition (ADAS-cog). Diagnosis of MCI was according to the Petersen criteria and consisted of patients showing a CDR 0.5+ and an MMSE value from 20 to 24 (Petersen et al., 2001). Diagnosis of AD was confirmed with a CDR 1+ and as defined according to the Diagnostic and Statistical Manual of Mental Disorders - fourth edition (DMS-IV). Clinical diagnosis of probable AD or MCI was made according to the 2011 Criteria and Guidelines for Alzheimer’s Disease Diagnosis of the Alzheimer’s Association.

All AD and MCI patients underwent complete medical and neurological evaluations, laboratory analyses and CT or MRI scans to exclude reversible causes of their dementia. Standard laboratory tests performed at the time of diagnosis included complete blood count, serum electrolytes, serum glucose, blood urea nitrogen, thyroid function tests and serology for syphilis. After completion of their general and neurological examinations, blood samples were immediately collected.

Informed consent was obtained from all study subjects with these forms complying to the stipulations of the Declaration of Helsinki. For severely demented participants, the consent form was signed by their legal guardian. All protocols for this study were approved by the medical ethics committee of Harbin Medical University.

#### Inclusion and Exclusion Criteria

A total of 20 AD and 22 MCI patients were recruited over the period from November 2017 to July 2018 from clinical referrals to the Department of Neurology in the First Affiliated Hospital of Harbin Medical University. Inclusion criteria were: (1) age ≥ 60 years, (2) diagnosis of MCI or AD and (3) completion of the informed consent form. A total of 22 controls from the community were recruited with the inclusion criteria of: (1) age ≥ 60 years, (2) good health at the time of the interview and (3) absence of any diagnosis of dementia. Controls were also matched with AD cases for sex and age.

Subjects with any of the following conditions were excluded: (1) a history of intercurrent infections, concomitant or past inflammatory and/or autoimmune diseases or previously reported diseases in which serum levels of netrin-1 were probably affected, (2) a previous TIA/stroke and/or a history of cerebrovascular disease (3) severe congestive heart failure, kidney disease, or advanced COPD, (4) cancer or hematological tumors, (5) concomitant medical, neurological or psychiatric illness known to affect cognition or a significant head trauma (6) recent major surgery, (7) chronic or recent (within 24 h) intake of anti-inflammatory drugs (NSAID and steroids) or AD related medication and (8) a history of alcohol and/or drug abuse, or disturbed levels of consciousness (D’Anna et al., 2017).

### Sample Preparation and Data Acquisition

All participants were required to fast for 8 h after their last meal before blood sample collection. Serum samples were obtained via standard procedures, aliquoted in polypropylene tubes and immediately stored at –80°C. Netrin-1, IL-17 and TNF-α levels in serum were measured using commercially available enzyme-linked immunosorbent assay (ELISA) kits according to the manufacturers’ instructions. Human netrin-1, IL-17 and TNF-α kits were from CUSABIO (Wuhan, China), Abnova (Heidelberg, Germany) and R&D Systems, respectively. Serum samples were diluted at 1:2 for TNF-α or remained undiluted for netrin-1 and IL-17. Minimally detectable concentrations were 31.25 pg/ml for netrin-1, 0.05 pg/ml for IL-17 and 1.0 pg/ml for TNF-α and coefficients of variations were 4.2%, 4.1%, and 4.8%, respectively.

### Statistical Analysis

Statistical analysis was performed using the SPSS 21.0 program. Continuous variables were expressed as means ± standard deviations (SD) and categorical variables as percentages. Normality tests were performed with these data. An analysis of variance (ANOVA) with the LSD test for *post hoc* pairwise comparisons was used to evaluate differences among groups showing normal distributions. Comparisons of categorical variables were performed using the χ^2^ test. The Pearson test was used for assessing correlations. A *P* < 0.05 was required for results to be considered as statistically significant.

## Results

### Characteristics of Study Subjects

Demographic and clinical characteristics of the study subjects are presented in Tables 1, 2. There were no statistically significant differences in sex or age among the MCI, AD and control subjects. MMSE scores were 14.20 ± 4.04 in AD, 22.00 ± 1.30 in MCI and 26.73 ± 1.61 in the control group. No cases were removed during our study, and all human information of each individual case has been listed in the Supplementary Tables.

**TABLE 1:** Demographic information of the study subjects.

	n	Sex[*n*(%)]		Age(years)	MMSE
		Female	Male		
AD	20	11(55.0)	9(45.0)	70.75 ± 7.32	14.20 ± 4.04
MCI	22	12(54.5)	10(45.5)	66.91 ± 5.36	22.00 ± 1.30
Controls	22	13(59.1)	9(40.9)	68.73 ± 6.79	26.73 ± 1.61
*F*/χ^2^	-	0.111		1.820	-
*P*	-	0.946		0.171	-
					

**TABLE 2:** Clinical features of the study subjects.

	n	Serum Netrin-1 levels (pg/ml)	Serum IL-17 levels (ng/ml)	Serum TNF-α levels (pg/ml)
AD	20	492.80 ± 135.51	68.06 ± 17.49	872.88 ± 288.41
MCI	22	553.33 ± 94.24	56.51 ± 9.34^a^	727.54 ± 188.65^a^
Controls	22	656.17 ± 161.41^ab^	41.50 ± 14.65^ab^	414.99 ± 146.51^ab^
*F*/χ^2^	-	8.133	18.754	25.526
*P*	-	0.001	<0.001	<0.001

*^a^ versus AD,^b^ versus MCI, P < 0.05.*

### Serum Netrin-1 Levels and Correlations With Mini Mental State Examination Scores

**FIGURE 1:**
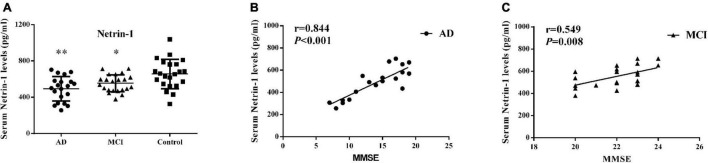
Serum netrin-1. **(A)** Concentrations of netrin-1 in AD patients, MCI patients and controls. Bar graphs represent the mean ± SD, ^∗^*P* ≤ 0.05 and ^∗∗^*P* ≤ 0.01 versus controls. Correlations between serum netrin-1 levels and MMSE scores in **(B)** AD and **(C)** MCI patients.

As presented in Figure 1A and Table 2, serum netrin-1 levels showed a statistically significant decrease in AD (492.80 ± 135.51 pg/ml, *P* < 0.001) and MCI (553.33 ± 94.24 pg/ml, *P* = 0.013) patients as compared with controls (656.17 ± 161.41 pg/ml). Serum netrin-1 levels and the MMSE scores showed a robust positive correlation in both AD (*r* = 0.844, *P* < 0.001) and MCI (*r* = 0.549, *P* = 0.008) patients (Figures 1B,C).

### Serum IL-17 Levels and Correlations With Mini Mental State Examination Scores

**FIGURE 2:**
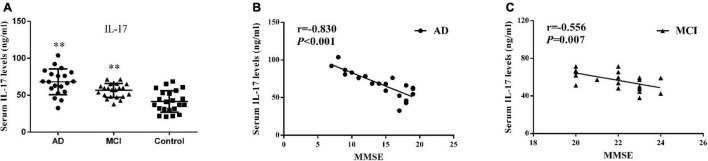
Serum IL-17. **(A)** Concentrations of IL-17 in AD patients, MCI patients and controls. Bar graphs represent the mean ± SD, ^∗∗^*P* ≤ 0.01 versus controls. Correlations between serum IL-17 levels and the MMSE scores in **(B)** AD and **(C)** MCI patients.

As shown within Figure 2A and Table 2, serum IL-17 levels were significantly increased in AD (68.06 ± 17.49 ng/ml, *P* < 0.001) and MCI (56.51 ± 9.34 ng/ml, *P* = 0.003) patients as compared with controls (41.50 ± 14.65 ng/ml). A strong negative correlation was present between serum IL-17 levels and MMSE scores in AD (*r* = –0.830, *P* < 0.001) and MCI (*r* = –0.556, *P* = 0.007) patients (Figures 2B,C).

### Serum TNF-α Levels and Correlations With Mini Mental State Examination Scores

**FIGURE 3:**
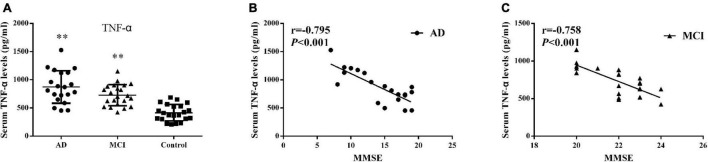
Serum TNF-α **(A)** Concentrations of TNF-α in AD patients, MCI patients and controls. Bar graphs represent the mean ± SD, ^∗∗^*P* ≤ 0.01 versus controls. Correlations between serum TNF-α levels and the MMSE scores in **(B)** AD and **(C)** MCI patients.

Detectable levels of serum TNF-α in the 3 groups are presented in Figure 3A and Table 2. Both AD (872.88 ± 288.41 pg/ml, *P* < 0.001) and MCI (727.54 ± 188.65 pg/ml, *P* < 0.001) patients showed significantly greater concentrations of serum TNF-α than that obtained in controls (414.99 ± 146.51 pg/ml). Serum TNF-α levels and MMSE scores showed a strong negative correlation in AD (*r* = –0.795, *P* < 0.001) and MCI (*r* = –0.758, *P* < 0.001) patients (Figures 3B,C).

### Correlations Among Levels of Serum Netrin-1, IL-17 and TNF-α

**FIGURE 4:**
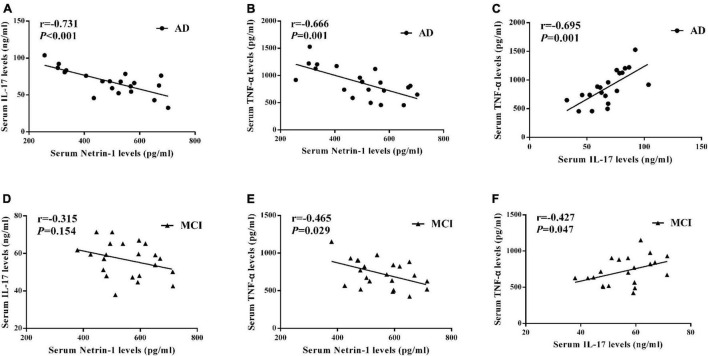
Correlations among serum **(A)** netrin-1 and IL-17 in AD patients, **(B)** netrin-1 and TNF-α in AD patients, **(C)** IL-17 and TNF-α in AD patients, **(D)** netrin-1 and IL-17 in MCI patients, **(E)** netrin-1 and TNF-α in MCI patients, and **(F)** IL-17 and TNF-α in MCI patients.

A negative correlation was present between serum netrin-1 and IL-17 levels in AD patients as shown in Figure 4A (*r* = –0.731, *P* < 0.001). Although there was a trend toward a negative correlation between serum netrin-1 and IL-17 levels in MCI patients, these results failed to achieve statistical significance (Figure 4D, *r* = –0.315, *P* = 0.154).

We also found that serum netrin-1 levels were negatively correlated with TNF-α levels in both AD (Figure 4B, *r* = –0.666, *P* = 0.001) and MCI (Figure 4E, *r* = –0.465, *P* = 0.029) patients. A positive correlation was present between serum TNF-α and IL-17 levels in both AD (Figure 4C, *r* = 0.695, *P* = 0.001) and MCI (Figure 4F, *r* = 0.427, *P* = 0.047) patients.

## Discussion

It is generally accepted that neuroinflammation plays a critical role in the pathogenesis and development of AD. This follows from the findings of several studies which have reported that a variety of inflammatory cytokines are present within AD patients. However, the change of serum netrin-1, along with the association among netrin-1, cognitive impairment and serum cytokines (IL-17 and TNF-α) in AD/MCI patients remains unclear. Here we present the first evidence that serum concentrations of netrin-1 are significantly decreased in AD patients, effects which were correlated with MMSE scores. These MMSE score provides a brief and objective screening tool for assessing cognitive impairment, with low scores indicating both the likelihood of cognitive impairment and need for further evaluation. In contrast to that of netrin-1, we found that serum levels of IL-17 and TNF-α were elevated in AD patients and negatively correlated with MMSE scores in the present study. These findings are in accord with previous studies and support the concept that increases in serum IL-17 and TNF-α concentrations play a role in AD (Holmes et al., 2009; Swardfager et al., 2010; D’Anna et al., 2017). In addition, we demonstrate that netrin-1 levels are inversely correlated with TNF-α in AD cohorts, as well as with IL-17.

Mild cognitive impairment (MCI) has been widely considered as transitional zone between normal aging and dementia. Mild cognitive impairment individuals with low-grade cognitive deficits are generally able to complete normal activities. Mild cognitive impairment can be divided into two forms: amnestic MCI and non-amnestic MCI. Memory deficits are remarkable in amnestic MCI cohorts, while other cognitive impairments, consisting of diminished attention, language or executive capability characterize non-amnestic MCI cohorts (Dugger et al., 2015). Amnestic MCI patients may have a higher risk of developing AD, while non-amnestic MCI patients may have a higher chance of developing other types of dementia, such as frontotemporal dementia or dementia with Lewy bodies (Geda, 2012; Roberts and Knopman, 2013). We also observed that serum netrin-1 levels were decreased in MCI patients and were positively correlated with MMSE scores, while serum concentrations of IL-17 and TNF-α were increased in MCI patients and were negatively correlated with MMSE scores. Levels of netrin-1, but not IL-17, were inversely correlated with TNF-α in MCI subjects. Although an inverse trend was observed between serum netrin-1 and IL-17 in MCI cohorts, these results failed to achieve statistical significance. Overall, reports demonstrating correlations between netrin-1 and other inflammatory cytokines are rare. Our present study also provides the first evidence indicating that netrin-1 may participate in the pathogenesis of MCI and, may even contribute to the transition from MCI to AD. Further studies with larger samples will be required to substantiate these relationships between netrin-1 and inflammatory cytokines in MCI.

Netrin-1 is expressed in the adult vertebrate nervous system as well as several non-neuronal tissues, such as, otic epithelium (Salminen et al., 2000), pancreas (Breuck et al., 2003; Yebra et al., 2003), lung (Dalvin et al., 2003; Liu et al., 2004) and mammary gland (Srinivasan et al., 2003). Within the nervous system, netrin-1 is secreted by floor plate cells, diffuses in the extracellular matrix and then stimulates the growth of commissural axons which extend toward the ventral midline (Serafini et al., 1994; Tessier-Lavigne and Goodman, 1996). In contrast to the long-range function in the embryonic nervous system, in the adult mammalian central nervous system (CNS), netrin-1 is expressed by oligodendrocytes, the myelinating cells of the CNS. A down-regulation of netrin-1 has also been observed in inflammatory and autoimmune diseases, such as multiple sclerosis (Mulero et al., 2017), type 2 diabetes mellitus patients (Liu et al., 2016), aneurysmal subarachnoid hemorrhage (Chen J.-L. et al., 2019), atherosclerosis (Bruikman et al., 2020) and renal ischemia-reperfusion injury (Tadagavadi et al., 2010). In Aβ1-42-induced rat model of AD, netrin-1 concentrations were seen to reduce in both serum and CSF (Sun et al., 2019). Furthermore, exogenous application of netrin-1 has been found to regulate Aβ peptide production, oligomerization and clearance in AD models, to improve cognitive dysfunction as assessed in the novel object recognition task and ameliorate impaired spatial memory as demonstrated in the morris water maze test (Lourenço et al., 2009; Spilman et al., 2016; Shabani et al., 2017; Zamani et al., 2019). Previous data also indicates that down-regulation of netrin-1 attribute to hyper-methylation of its gene can result in memory loss (Kalani et al., 2019). In addition, netrin-1 protected Aβ1-42-exposed SH-SY5Y cells through NF-κB/Nrf2 dependent mechanism (Zamani et al., 2020). Netrin-1 receptor UNC5C has also been proven an AD risk gene previously (Wetzel-Smith et al., 2014; Chen et al., 2021).

As mentioned above, it has been demonstrated numerous inflammatory cytokines are associated with AD. These data point to a putative role for their capacity to induce and maintain inflammation within the CNS and, in specific, AD. Netrin-1 also confers anti-inflammatory effects through these biomarkers as demonstrated in a number of cellular and animal models of disease. For example, netrin-1 protected against acute lung injury sepsis in rats through decreasing the expression of IL-1, IL-6, and TNF-α (Liu et al., 2019), and in a porcine model of acute lung injury, through ameliorating TNF-α, IL-1β, IL-6 and IL-8 (Mutz et al., 2010). Netrin-1 has also been shown to regulate inflammatory cytokine production (IL-2, IL-6, IL-10, IL-13, IL-17, IFN-γ, IL-4, and TNF-α) (Tadagavadi et al., 2010) as well as inflammatory responses of macrophages (Ranganathan et al., 2013a) in renal ischemia-reperfusion injury and its associated renal inflammation. It seems likely that at least part of these effects of netrin-1 may be mediated through suppression of STAT3 and JNK signaling and IL-6 expression (Ranganathan et al., 2013b). Netrin-1 also modulates colon-kidney cross talk through reducing both the production and activity of IL-6 as demonstrated in a mouse model of DSS-colitis (Ranganathan et al., 2013). Treatment of corneal Aspergillus fumigatus infections in mice with exogenous netrin-1 attenuates the inflammatory response by reducing IL-1β and TNF-α and up-regulating IL-10 (Zhou et al., 2019). Within our laboratory, we have demonstrated that netrin-1 concentrations were negatively correlated with IL-17 but positively correlated with IL-10 concentrations in the serum and CSF of AD rats, effects which appear to involve a disruption in the Th17/Tregs balance (Sun et al., 2019). Moreover, in this study, we also found that serum netrin-1 levels were inversely related with IL-17 levels in AD patients. The cytokine IL-10 is a key anti-inflammatory mediator involved with limiting neuronal damage during infection or other inflammatory processes, as well as being implicated in regulating homeostatic processes (Knoblach and Faden, 1998; Grilli et al., 2000; Zhou et al., 2009a,b). Therefore, the decreases in netrin-1 as observed in both AD patients and animal models of AD and resultant loss of inhibition in the production of pro-inflammatory cytokines may represent a significant contributing factor for AD. We have hypothesized that, at least a part of the protective effects of netrin-1 in AD may involve regulating IL-10 production. However, there also exist findings from some studies which do not support a relationship between IL-17 and AD (Saresella et al., 2011), or TNF-α and AD (D’Anna et al., 2017). Such contradictory results may be explained by the biological variability and different sizes and samples of AD patients investigated. Although our current results suggest a relationship of these cytokines with AD, their exact roles in producing AD have yet to be thoroughly investigated.

Recent findings on proteomic profiles of samples from both human (cortex, CSF and serum) and 5xFAD mice (cortex) have revealed a number of novel AD biomarker candidates, of which netrin-1 represented one of the most prominent proteins (Bai et al., 2020; Wang et al., 2020). Results as obtained through procedures involving direct Aβ-binding (Borel et al., 2017), have indicated that netrin-1 was highly colocalized with Aβ in amyloid plaques, suggesting that they may function together within the brains of AD patients and mouse models of AD (Bai et al., 2020). However, as related RNA levels of netrin-1failed to show a statistically significant increase with RNA-seq analysis, it appears that netrin-1 was regulated by posttranscriptional mechanisms in this 5xFAD mouse model (Bai et al., 2020). In our study, netrin-1 levels were decreased in AD and MCI patients (serum), as well as in the Aβ1-42-induced rat model of AD (serum and CSF). As netrin-1 was found to be highly correlated with Aβ, it seems possible that plaque development slows down the turnover of netrin-1, which at least partially contributes to its accumulation in the AD brain. Such a process may induce a compensatory feedback that could attenuate the toxic Aβ insult attributable to the initial accumulation of Aβ in AD whereas, in the absence of this process, additional factors may be required to interrupt the vicious cycle that leads to irreversible degeneration. After an intracerebroventricular administration of Aβ1-42 for 7 days, amyloid plaques were observed in the cortex and hippocampus of this rat model of AD (Yaghmaei et al., 2014). Within this same AD rat model, both serum and CSF concentrations of netrin-1 were decreased (Sun et al., 2019), while an exogenous application of netrin-1 was found to regulate Aβ peptide production, oligomerization and clearance in animal models of AD (Lourenço et al., 2009; Spilman et al., 2016; Shabani et al., 2017; Zamani et al., 2019). Decreases in CSF Aβ1-42 are associated with a cascade of events consisting of fibrillar Aβ deposition (positive amyloid-PET), increased tau in the CSF (CSF tau), hippocampal atrophy and hypometabolism and finally cognitive and clinical deficits in the later stages (Bateman et al., 2012). In general, a down-regulation of CSF Aβ1-42 (Bateman et al., 2012), which then leads to increased Aβ deposition (Fagan et al., 2007), precedes other AD-related biomarker changes in humans by years. Here, we present the first evidence that decreased serum netrin-1 levels in AD and MCI patients, are positively correlated with cognitive deficits. Therefore, it seems likely that low CSF Aβ1-42, Aβ deposition (positive amyloid-PET) and decreased serum netrin-1 levels may be simultaneously present in AD and MCI patients. Future studies will be required to reveal the details of these relationships among netrin-1, Aβ (serum or CSF) and Aβ PET scan in the brains of AD patients.

We are aware of limitations in this study, especially with regard to sample size. This small sample size was due to the application of a more selective exclusion criteria of subjects than that typically employed in other studies. This stringent selection process enabled us to achieve a high degree of reliability with regard to our cytokine evaluations. Reliability of cytokine evaluations represents an important issue which can be strongly influenced by confounding factors such as other pathological conditions and concomitant medications. As patients and controls rarely agreed to undergo CSF collection it was not possible to determine if inflammatory cytokines in serum (netrin-1, IL-17 and TNF-α) are correlated with specific CSF biomarkers of AD. Future studies with larger sample sizes will be required to validate our results. Moreover, an evaluation of the diagnostic value of netrin-1 as combined with amyloid pathology/neurodegeneration blood biomarkers (e.g., Aβ, Tau and phosphorylated Tau) and amyloid-PET findings would provide further pertinent information regarding their use and validity as AD markers. Future studies with larger sample sizes and inclusion of additional inflammatory markers for assessment will be required to validate our results. Additional information on follow-up visits at set intervals would also be beneficial for this investigation. Finally, molecular studies directed at providing a better understanding of the mechanisms of netrin-1 within the AD injury process would be needed to establish a direct relationship between this neuronal cue and cognitive status.

## Conclusion

In summary, compared to controls, serum netrin-1 levels were diminished in AD and MCI patients and were positively correlated with MMSE scale scores. Like that as reported in previous studies, serum IL-17 and TNF-α levels were elevated in AD and MCI cohorts and were negatively correlated with MMSE scale scores. Serum netrin-1 levels were inversely correlated with IL-17 and TNF-α levels in AD, but not MCI, patients. Based on the data reported here, we conclude that netrin-1 may be a promising biomarker that may enable a reliable and early diagnosis and prognosis of AD.

## Data Availability Statement

The raw data supporting the conclusions of this article will be made available by the authors, without undue reservation.

## Ethics Statement

Written informed consent was obtained from the individual(s) for the publication of any potentially identifiable images or data included in this article.

## Author Contributions

LC and TJ: study design and manuscript writing. YF and LS: data analysis. DL and YL: ELISA. TL, CZ, and WW: subjects’ recruitment. TW: manuscript revision. All authors contributed to the article and approved the submitted version.

## Conflict of Interest

The authors declare that the research was conducted in the absence of any commercial or financial relationships that could be construed as a potential conflict of interest.

## Publisher’s Note

All claims expressed in this article are solely those of the authors and do not necessarily represent those of their affiliated organizations, or those of the publisher, the editors and the reviewers. Any product that may be evaluated in this article, or claim that may be made by its manufacturer, is not guaranteed or endorsed by the publisher.
