# Supplementary material for: Decreased Netrin-1 in Mild Cognitive Impairment and Alzheimer’s Disease Patients
Source: Front Aging Neurosci. 2022 Feb 16;13:762649. doi: 10.3389/fnagi.2021.762649 (PMC8888826; doi:10.3389/fnagi.2021.762649)
Supplement: Supplementary file 1 [file Table_1.docx]

| No. | Sex  (F: Female; M: Male) | Age(years) | MMSE |
| --- | --- | --- | --- |
| 1 | M | 65 | 12 |
| 2 | F | 77 | 7 |
| 3 | F | 82 | 17 |
| 4 | F | 82 | 18 |
| 5 | F | 74 | 18 |
| 6 | F | 67 | 18 |
| 7 | M | 70 | 19 |
| 8 | F | 75 | 9 |
| 9 | F | 62 | 17 |
| 10 | M | 68 | 15 |
| 11 | M | 79 | 10 |
| 12 | F | 64 | 13 |
| 13 | M | 65 | 8 |
| 14 | F | 60 | 11 |
| 15 | M | 73 | 15 |
| 16 | M | 71 | 19 |
| 17 | M | 62 | 14 |
| 18 | F | 71 | 16 |
| 19 | F | 84 | 9 |
| 20 | M | 64 | 19 |

**Supplemental Table 1.** All human information of each AD individual case
