# Supplementary material for: Decreased Netrin-1 in Mild Cognitive Impairment and Alzheimer’s Disease Patients
Source: Front Aging Neurosci. 2022 Feb 16;13:762649. doi: 10.3389/fnagi.2021.762649 (PMC8888826; doi:10.3389/fnagi.2021.762649)
Supplement: Supplementary file 2 [file Table_2.docx]

| No. | Sex  (F: Female; M: Male) | Age(years) | MMSE |
| --- | --- | --- | --- |
| 1 | F | 68 | 24 |
| 2 | M | 65 | 23 |
| 3 | M | 66 | 22 |
| 4 | F | 62 | 21 |
| 5 | M | 65 | 23 |
| 6 | F | 60 | 22 |
| 7 | F | 68 | 20 |
| 8 | F | 70 | 23 |
| 9 | M | 62 | 20 |
| 10 | M | 68 | 22 |
| 11 | M | 74 | 24 |
| 12 | M | 71 | 23 |
| 13 | M | 61 | 22 |
| 14 | F | 75 | 22 |
| 15 | F | 76 | 20 |
| 16 | F | 73 | 23 |
| 17 | F | 67 | 22 |
| 18 | F | 60 | 23 |
| 19 | M | 61 | 20 |
| 20 | F | 65 | 23 |
| 21 | M | 60 | 22 |
| 22 | F | 75 | 20 |

**Supplemental Table 2.** All human information of each MCI individual case
