# Supplementary material for: Decreased Netrin-1 in Mild Cognitive Impairment and Alzheimer’s Disease Patients
Source: Front Aging Neurosci. 2022 Feb 16;13:762649. doi: 10.3389/fnagi.2021.762649 (PMC8888826; doi:10.3389/fnagi.2021.762649)
Supplement: Supplementary file 3 [file Table_3.docx]

| No. | Sex  (F: Female; M: Male) | Age(years) | MMSE |
| --- | --- | --- | --- |
| 1 | F | 72 | 25 |
| 2 | M | 60 | 29 |
| 3 | F | 61 | 27 |
| 4 | M | 71 | 28 |
| 5 | F | 62 | 26 |
| 6 | F | 71 | 25 |
| 7 | F | 77 | 25 |
| 8 | F | 62 | 25 |
| 9 | M | 72 | 25 |
| 10 | M | 79 | 26 |
| 11 | M | 80 | 29 |
| 12 | M | 65 | 29 |
| 13 | F | 62 | 28 |
| 14 | F | 68 | 27 |
| 15 | M | 63 | 28 |
| 16 | F | 79 | 25 |
| 17 | F | 60 | 25 |
| 18 | F | 75 | 28 |
| 19 | M | 61 | 29 |
| 20 | M | 71 | 26 |
| 21 | F | 74 | 28 |
| 22 | F | 67 | 25 |

**Supplemental Table 3.** All human information of each Control individual case
